# Performance of a North American Field Population and a Laboratory Colony of the Potato Tuberworm, *Phthorimaea operculella*, on Foliage of Resistant and Susceptible Potato Clones

**DOI:** 10.1673/031.010.8001

**Published:** 2010-06-29

**Authors:** Mahmut Doğramaci, Ward M. Tingey

**Affiliations:** ^1^ Cornell University, Department of Entomology, Ithaca, NY 14853; ^2^ Current address: University of Florida, Mid-Florida Research and Education Center, Apopka, FL 32703

**Keywords:** larval survival, potato breeding, potato resistance, potato tubermoth, *Solanum tuberosum*, *Solanum berthaultii*

## Abstract

Foliar resistance of two potato clones was tested against a Columbia Basin field population (CBFP) and a Colorado laboratory colony (COLC) of the potato tuberworm, *Phthorimaea operculella* (Zeller) (Lepidoptera: Gelechiidae). The first clone was a cross of a cultivated potato, *Solanum tuberosum* L. (Solanales: Solanaceae), and a wild potato, *Solanum berthaultii* Hawkes (Q 174-2); the second clone was cv. Allegany, *S. tuberosum* L.. In no-choice assays, defoliation by *P. operculella* larvae of COLC and CBFP did not differ on Allegany and Q174-2. Larval weight and production of COLC and CBFP colonies were similarly reduced on Q174-2 compared to cv. Allegany, although larval weights and production of the CBFP population were slightly less affected by the host. Larval production by the COLC on Allegany was greater than that on Q174-2, while that of the CBFP on Allegany and Q174-2 did not differ. However, production of *P. operculella* larvae by the CBFP on Q174-2 during no-choice assays was greater than that in choice tests, indicating reduced host preference. Most of the larvae recovered from either host were fourth instars, followed by third instars. Although the levels of resistance expressed by Q174-2 potato clone to the two *P. operculella* populations differed in magnitude, nearly all of *P. operculella* performance criteria measured in this study were adversely affected by Q174-2 foliage compared to the commercial potato cultivar, cv. Allegany.

## Introduction

The potato tuberworm, *Phthorimaea operculella* (Zeller) (Lepidoptera: Gelechiidae), is a destructive insect pest of solanaceous crops, especially cultivated potato, *Solanum tuberosum* L. (Solanales: Solanaceae), (Lal 1987; Sporleder et al. 2004). Severity of the pest mainly depends upon its ability to retain large numbers in winter (Lal 1987). *P. operculella* is considered the most damaging insect pest of potatoes in developing countries in the tropics and subtropics regions (Fenemore 1977, 1980, 1988). *P. operculella* was first detected in the United States in California in 1856, and since then it has been a pest of potatoes in southern California and other southern states (Graf 1917). *P. operculella* does not have true diapause and can produce as many 18 generations in a year when environmental conditions in the field and in non-refrigerated storages are favorable (Kabir 1994).

Although *P. operculella* has been recorded in many parts of the United States, it has not been considered a major potato pest in the northern states (Graf 1917; Bacon 1960) until recently, when it was found responsible for significant losses in the Columbia Basin (Rondon et al. 2007), apparently because of its ability to survive winter conditions in the Oregon production region (Doğramaci et al. 2008b). The Columbia Basin region (Pacific Northwest) produces about 55% of the total U.S. potato production, accounting for more than 46% of potato farm value in the U.S. (Araji and Love 2002). While adult *P. operculella* have been reported from surveillance traps in Idaho and Washington, significant field damage to potatoes in these areas has not yet been reported. However, the Columbia Basin constitutes a large percentage of potato production in the United States, and *P. operculella* poses a serious threat to potato production in that area. Females deposit eggs on foliage, and larvae cause defoliation by mining mesophyll layers. While defoliation may not cause significant yield losses, larval infestation of tubers in the field prior to harvest and in stored potatoes can affect marketability (Ojero and Mueko 1985; Valencia 1984). *P. operculella* can cause 50% yield loss associated with grade-outs when tuber infestation ≥ 4%; higher incidence of tuber infestation can render potatoes completely unmarketable in the U.S. (Columbia Basin potato growers and processors, 1996, personnel communication). Reduction of *P. operculella* populations on foliage will help in lowering later field tuber infestations that result in storage losses.

Chemical management of *P. operculella* is challenging because of the protected tunneling behavior of larvae in foliage and tubers and because this pest has developed resistance to many traditional organophosphate, carbamate, and pyrethroid insecticides (Bacon 1960; Shelton and Wyman 1979; Shelton et al. 1981; Collantes et al. 1986). *P. operculella* collected from potato foliage in the Columbia Basin were highly resistant to esfenvalerate (Asana®) (Doğramaci et al. 2008).

*Solanum berthaultii* Hawkes (Solanales: Solanaceae), a wild potato species possessing glandular trichomes, resists infestation by a wide range of potato pests including aphids (Gibson 1974; LePointe and Tingey 1984), potato leafhopper (Tingey and Laubengayer 1986; Medeiros and Tingey 2006, Mederios et al. 2004, 2005), and Colorado potato beetle (Casagrande 1982; Yencho and Tingey 1994). Studies have indicated that glandular trichomes of *S. berthaultii* entrap small, soft-bodied insects such as aphids and leafhoppers, causing starvation and death; glandular trichomes deter feeding by larger insects such as the Colorado potato beetle (Yencho and Tingey 1986). Glandular trichomes of *S. berthaultii* also produce a deterrent effect on oviposition and larval feeding and development of *P. operculella* (Musmeci et al. 1997; Malakar and Tingey 1999, 2000).

Glandular trichome traits of *S. berthaultii* have been incorporated into tuber-bearing potato clones resistant to the Colorado potato beetle and potato leafhopper through conventional plant breeding methods (Plaisted et al. 1992). The availability of *P. operculella*-resistant cultivars could be a promising tool for management of *P. operculella* in an environmentally sound manner (Raman and Palacios 1982). The objectives of the present study were two-fold: (1) to compare the development of *P. operculella* larvae from a Columbia Basin field population (CBFP) and a laboratory colony from Colorado (COLC) on foliage of resistant and susceptible potato clones and (2) to determine the influence of host foliage on several behavioral and developmental characteristics of *P. operculella*.

## Materials and Methods

### Plant culture

Potato germplasm used in this study included the commercial insect susceptible cultivar, cv. Allegany and a PTW resistant clone, Q174-2. The latter is a cross of cultivated potato, *S. tuberosum* L., and the wild, tuber-bearing species, *S. berthaultii* Hawkes. Q174-2 has been widely studied (Malakar-Kuenen and Tingey 2006; Medeiros and Tingey 2006; Mederios et al. 2004, 2005). Plants of Allegany and Q174-2 were grown from vegetative seed provided by Dr. Walter de Jong, Department of Plant Breeding and Genetics, Cornell University, Ithaca, NY. Seeds were individually planted in 25 cm diameter pots with peat-perlite soil mix (Pro-Mix BX, Premier Horticulture Inc., www.premierhort.com), fertilized weekly with 20-10-20 (N-P-K), irrigated as necessary, and grown in a greenhouse for four weeks at 26 ± 2° C with a photoperiod of 16:8 L:D. Natural lighting of the greenhouse was supplemented by 1000 watt metal halide multi vapor lamps yielding a maximum of 1000 µE m^-2^ s^-1^, and then plants were transferred to cages for assays. Cages were prepared from clear plastic storage boxes (Stacks & Stacks, www.stacksandstacks.com); the dimensions were 34 × 45 × 62 cm, and they had two ventilation openings (each 20 cm in diameter) on opposite sides. One of the openings was covered with screening fabric mesh and the other opening was covered with a fine polyester mesh sleeve (JoAnn Fabrics & Crafts, www.joann.com).

### Insect colonies

The potato tuberworm colonies used in this study were derived from a population of *P. operculella* maintained in a mass rearing environment (> 30 years) at the Colorado State Department of Agriculture, Palisade, CO, which was originally initiated with *P. operculella* collected from California. Another *P. operculella* colony was initiated from larvae collected from infested potato foliage from the Columbia Basin. Pre-pupa larvae were removed from the potato foliage, and the larvae were placed in a polyethylene food container containing fine silica sand for pupation environment. Voucher specimens of the parental *P. operculella* populations were deposited in the Cornell University Insect Collection under C.U. lot number 1254. The two colonies were held in separate environmental rearing chambers throughout this study to prevent intermixing. Each colony was propagated and maintained by placing approximately five pairs (male and female) of *P. operculella* in each of approximately 10 polystyrene boxes (18 × 14 × 95 cm), (Pioneer Plastic, www.pioneerplastic.com) each containing from four to five tubers of cv. Allegany. The rearing boxes were held in environmental growth chambers at 26 ± 1° C with 24 h scotophase. Females deposited eggs on the tubers, and the hatched neonates were allowed to feed on the same tubers for full term larval development. Pre-pupa larvae were transferred into 473 ml polyethylene food containers (Reynolds Del-Pak Bulk Pack Container, Paper House Inc., www.paperhouse.com) containing fine silica sand as pupation substrate. The pupation containers were held in growth chambers at 26 ± 1° C with 24 h scotophase. The three- to four-day-old pupae were separated from the sand by sieving and placed singly in 000 size gelatin capsules (Natural/Natural Capsules, Capsuline, www.capsuline.com) to await eclosion and sex determination.

### Measurement of larval development and foliage feeding

**No-choice assay.** Plants of Q174-2 and Allegany grown in the greenhouse were individually placed in clear plastic box cages and transferred into a room at 24 ± 2° C, 40–50% RH with a photoperiod of 16:8 L:D. Room lighting was supplemented by 40 watt, Sylvania Gro-Lux. One female and two male *P. operculella* from either CBFP or COLC were placed on the caged plants until deceased. Larvae were allowed to feed and develop on each caged plant; the assay was terminated when defoliation of at least one of the replicates reached 70% or when pre-pupal larvae were first observed. Defoliation was assessed by visually comparing damaged area of leaves to the total leaf area, after which all foliage was excised at the soil surface and dissected to recover *P. operculella* larvae in and on leaves and stems. Data were recorded as percent defoliation, numbers, and individual weight of larvae. The assay was arranged as a factorial design of two factors (2 potato varieties × 2 potato tuberworm colonies) with five replicates. PROC GLM procedure was used to test variances, and means that were significantly different at α = 0.05 were compared using a least significant difference (LSD) test (SAS Institute, 1997). To evaluate the correlation between *P. operculella* PTW colonies and potato clones, Pearson's correlation was used.

**Choice assay.** This assay was processed similar to the no-choice assay except for changes in host plants and numbers of adult *P. operculella* per cage. One plant each of Q174-2 and Allegany and two female and four adult male *P. operculella* were placed in each cage. The adults were kept on caged plants until deceased. Larvae were allowed to feed and develop on each caged plant; the assay was terminated when defoliation of at least one of the replicates reached 70% or when pre-pupal larvae were first observed. Defoliation was assessed by visually comparing damaged area of leaves to the total foliage of the plants, after which all foliage was excised at the soil surface and dissected to recover larvae in and on leaves and stems. Data were recorded as percent defoliation, numbers, and individual weight of larvae. The assay was arranged as a factorial design of two factors (2 potato variety × 2 potato tuberworm colonies) with 10 replications. PROC GLM procedure was used to test variances, and means that were significantly different at α = 0.05 were compared using a least significant difference (LSD) test (SAS Institute, 1997).

**Larval instar.** To examine the influence of the host on larval development larvae recovered from the foliage were weighed and placed in 90% alcohol for measurement of head capsules. Ten larvae from each replication were selected for head capsule measurement; head capsules were severed from the thorax using a razor blade and positioned under a stereomicroscope for accurate measurement. Width and length of the head capsules were measured using an eyepiece ocular micrometer with units of 12.5 µm at 80x magnification. Larvae were classified as first instar if the width of head capsule was < 0.304 mm, second instar if the width of head capsule was > 0.304 and < 0.357, third instar if the width of head capsule was > 0.360 and < 0.725 mm, and fourth instar if the width of head capsule was > 0.726 mm (Broodryk 1970; Chauhan and Verma 1990). Data were analyzed as a factorial design of two factors (2 potato variety × 2 potato tuberworm colonies). Each instar was analyzed separately. PROC GLM procedure was used to test variances, and means that were significantly different at α = 0.05 were compared using a least significant difference (LSD) test (SAS Institute, 1997).

**Figure 1.**
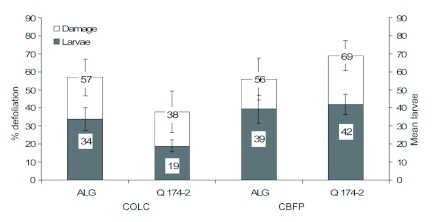
Comparison of foliage feeding by larval *Phthorimaea operculella* and larval production (mean ± SE) on *Solanum berthaultii* clones Allegany and Q174-2 for the COLC and CBFP colonies. High quality figures are available online.

## Results

### Larval development and foliar feeding

**No-choice assays.** Interaction between tuberworm colonies and potato clones in regard to foliar damage was not significant (*F* = 2.34; df = 1, 35; p = 0.13). Colony origin did not affect leaf damage; larvae from COLC and CBFP produced similar levels of defoliation (*F* = 2.06; df = 1, 35; p = 0.16) on both Allegany and Q174-2 (Figure 1). Although defoliation on Allegany and Q174-2 did not differ (*F* = 0.09; df = 1, 35; p = 0.76), fewer larvae from COLC were recovered (*F* = 5.29; df = 1, 35; p = 0.02) than from CBFP. Greater numbers of COLC larvae developed on Allegany than on Q174-2, while numbers of CBFP larvae produced on Q174-2 were similar to those on cv. Allegany, indicating minimal impact of Q174-2 on the survival of larvae from the CBFP colony. The interaction between tuberworm colony and potato cultivar in regard to numbers of larvae was not significant (*F* = 2.08; df = 1, 35; p = 0.15). The correlation between defoliation and numbers of larvae recorded on Allegany and Q174-2 was significant (r = 0.95; p = 0.05) indicating that feeding damage increased, as expected, with numbers of larvae. The interaction between tuberworm colony and potato cultivar in regard to larval weight was not significant (*F* = 0.11; df = 1, 35; p = 0.74) indicating both of the colonies responded similarly when reared on Allegany and Q174-2. Larvae originating from each source colony (COLC and CBFP) were of similar weight (*F* = 2.30; df = 1, 35; p = 0.13), but those feeding on Q174-2 weighed significantly less than larvae reared on cv. Allegany (*F* = 3.88; df = 1, 35; p = 0.05) (Figure 3).

**Figure 2.**
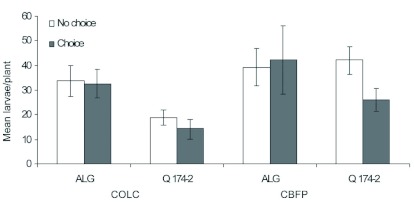
Mean numbers of *Phthorimaea operculella* larvae (mean ± SE) recovered from foliage of *Solanum berthaultii* clones Allegany and Q174-2 in choice and no-choice assays. High quality figures are available online.

**Figure 3.**
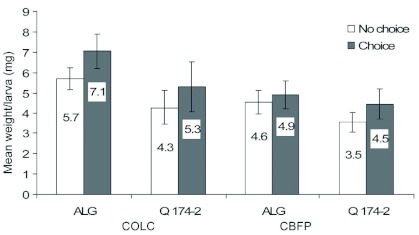
*Phthorimaea operculella* larval weight (mean ± SE) of COLC and CBFP on foliage of *Solanum berthaultii* clones Allegany and Q174-2 in choice and no-choice assays. High quality figures are available online.

**Choice assay.** Given the choice of hosts, both tuberworm colonies caused significantly greater foliar damage on Allegany than on Q174-2 (*F* = 8.27; df = 1, 35; p = 0.0068). Interaction between the colonies and clones in regard to foliar damage was not significant (*F* = 0.27; df = 1, 35; p = 0.6068). Significantly greater numbers of COLC and CBFP larvae were recovered from the foliage of Allegany than that of Q174-2 (*F* = 4.34; df = 1, 35; p = 0.04) (Figure 2). Total numbers of COLC larvae reared on Allegany and Q174-2 were not different than those of CBFP larvae reared on Allegany and Q174-2 (*F* = 1.85; df = 1, 35; p = 0.1822). Similar to foliar damage, interaction between the colonies and clones in regard to numbers of larvae was not significant (*F* = 0.02; df = 1, 35; p = 0.8966). Although larval weight of the COLC and CBFP reared on Allegany and Q174-2 did not differ significantly (*F* = 1.49; df = 3, 35; p = 0.2300), the trend was similar to that observed in the no-choice assays. There was no significant interaction between the colonies and clones in regard to larval weight (*F* = 0.49; df = 3, 35; p = 0.4882). The origins of the colonies had insignificant impact on larval weight (*F* = 2.75; df= 3, 35; p = 0.1061) that developed on both of the hosts. Mean larval weights of COLC and CBFP were 5.64 and 4.88 mg on Allegany and Q174-2, respectively when the assays were terminated (Figure 3).

**Figure 4.**
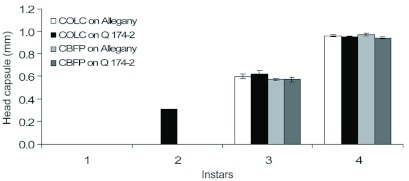
*Phthorimaea operculella* larval head capsule width (mean ± SE) of COLC and CBFP reared on foliage of *Solanum berthaultii* clones Allegany and Q174-2. High quality figures are available online.

**Larval instars.** Fourth instars comprised 78–92%) of recovered larvae, and only 8–22% were third instars (Table 1); no first instars were found, and the only second instars observed were two larvae of the COLC colony recovered from the foliage of Q174-2 (Figure 4). Third instars of the COLC colony had significantly larger head capsules than those of the CBFP population (*F* = 5.09; df = 1, 18; p = 0.02); head capsules were similar on Allegany and Q174-2 (*F* = 0.46; df= 1, 18; p = 0.50), and the interaction between the colonies and clones was not significant (*F* = 0.57; df = 1, 18; p = 0.45). Similarly interaction at fourth instars was also not significant (*F* = 0.32; df = 1, 18; p = 0.57). Host influence on head capsule size was not significant at the fourth stadium (*F* = 2.35; df = 1, 40; p = 0.13). Significant influence of colonies on head capsule at third instars diminished at fourth instars (*F* = 0.03; df = 1, 40; p = 0.86).

## Discussion

Foliage of the resistant hybrid potato, Q174-2, was generally inferior to that of the commercial cv. Allegany for performance of *P. operculella* larvae derived from the COLC and CBFP populations. Defoliation produced by COLC and CBFP larvae corresponded directly to their numbers on Allegany and Q174-2. However, fewer larvae were produced on foliage of Q174-2 than on that of Allegany, suggesting a deterrent effect on oviposition and/or larval establishment in this germplasm, as previously reported by Malakar and Tingey (1999) and Malakar-Kuenen and Tingey (2006).

Regardless of host or tuberworm origin, most of the recovered larvae were nearly full term fourth instars. A slightly greater proportion of third instars were produced on Q174-2 compared to Allegany, indicating possible nutritional or antibiotic resistance in foliage of Q174-2 (Malakar and Tingey 2000). While COLC larvae had larger head capsules than CBFP larvae at the third stadium, head capsules of fourth instars were of similar size, indicating a possible diminished influence of the host plant late in larval development.

Larval weights were generally similar in no-choice and choice assays for tuberworms from both sources and hosts, although CBFP larvae consistently weighed less than COLC larvae. Weights were consistent with those recorded on tubers of Allegany and Q174-2 (Doğramaci and Tingey 2008, unpublished data). Malakar and Tingey (1999) showed that oviposition deterrence against potato tuberworm could be mechanically transferred by pressing leaflets of *S. berthaultii* against foliage of susceptible potato varieties. They also reported that while some neonates successfully penetrated leaves of *S. berthaultii*, tunneling was significantly slowed, possibly because of pre-penetration encasement of tarsi by exudates from type A and B glandular trichomes.

Foliage of Q174-2 appears to possess resistance to *P. operculella* larval performance but certainly not at the level of its resistant parent, *S. berthaultii*, which produced consistent delays in larval development and reduced larval weight (Malakar and Tingey 1999). Reduced tunneling capability of larvae in foliage of Q174-2 might be associated with biochemical factors in Q174-2 leaves as well as defensive phenomena such as encapsulated tarsi as reported by Malakar-Kuenen and Tingey (2000).

**Table 1.**
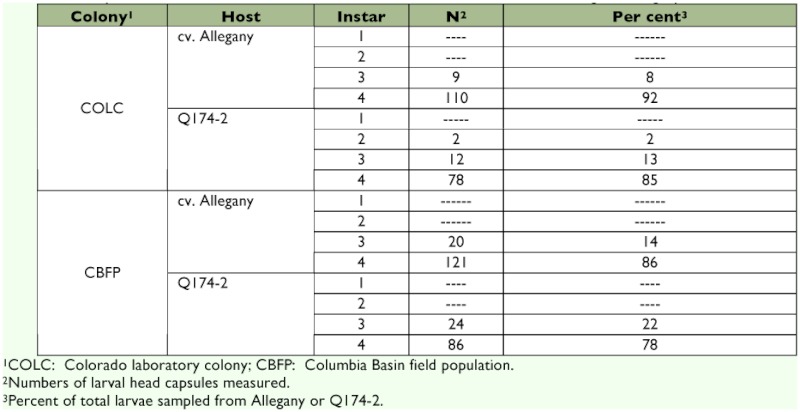
Comparison of PTW larval instars numbers of COLC and CBFP reared on foliage of Allegany and Q174-2.

In conclusion, foliage of Q174-2 expressed significant resistance against the COLC population and hindered ovipositional and/or developmental performance of the CBFP population, although the magnitude of the negative host influence was less on CBFP than that on COLC. These findings indicate that potentially useful levels of resistance exist in the foliage of a hybrid potato derived from a highly resistant wild species, and that the expression of resistance varies depending on the source of the *P. operculella* population. This information will be useful in future investigations of the genetic basis for resistance and in the development and utilization of varietal resistance for sustainable integrated management of PTW.

## Abbreviations

CBFP: Columbia Basin field *P. operculella* population
COLC: Colorado laboratory *P. operculella* colony
